# Quantifying the arms race between LINE-1 and KRAB-zinc finger genes through TECookbook

**DOI:** 10.1093/nargab/lqad078

**Published:** 2023-09-06

**Authors:** Zheng Zuo

**Affiliations:** Shenzhen University, Shenzhen, China

## Abstract

To defend against the invasion of transposons, hundreds of KRAB-zinc finger genes (ZNFs) evolved to recognize and silence various repeat families specifically. However, most repeat elements reside in the human genome with high copy numbers, making the ChIP-seq reads of ZNFs targeting these repeats predominantly multi-mapping reads. This complicates downstream data analysis and signal quantification. To better visualize and quantify the arms race between transposons and ZNFs, the R package TECookbook has been developed to lift ChIP-seq data into reference repeat coordinates with proper normalization and extract all putative ZNF binding sites from defined loci of reference repeats for downstream analysis. In conjunction with specificity profiles derived from *in vitro* Spec-seq data, human ZNF10 has been found to bind to a conserved ORF2 locus of selected LINE-1 subfamilies. This provides insight into how LINE-1 evaded capture at least twice and was subsequently recaptured by ZNF10 during evolutionary history. Through similar analyses, ZNF382 and ZNF248 were shown to be broad-spectrum LINE-1 binders. Overall, this work establishes a general analysis workflow to decipher the arms race between ZNFs and transposons through nucleotide substitutions rather than structural variations, particularly in the protein-coding region of transposons.

## INTRODUCTION

Most of human KRAB-zinc finger genes (ZNFs) have been identified as being involved in the recognition and silencing of specific repeat families, such as LINE, SINE, ERV, and DNA transposons (1–3). In a few known instances, including LINE-1, SVA and ERVL-MaLR (4–6), some relatively younger repeat members have evaded ZNFs' silencing through mutations, gaining a selective advantage for certain periods of time. This phenomenon is referred to as the arms race between transposons and ZNFs (7). Understanding the co-evolution or arms race between transposons and zinc finger genes (ZNFs) not only sheds light on our own evolutionary history but also reveals other biological functions of long ZNFs (8,9).

Some young transposons, such as the LINE-1 family member L1HS, are no more than 10 million years old and exhibit very limited sequence diversity between different genomic instances (Kimura Divergence of L1HS_5end: 3.8%, Source: Dfam (10)). This presents two unique challenges for data analysis and interpretation. Firstly, the motif discovery of many long ZNFs based on ChIP-seq data is difficult. For numerous ZNFs, including ZNF10, ZNF430, ZNF675 and others, most of their top ChIP-seq peaks are located within specific repeat families, and the sequence content within those peaks is highly similar, sometimes even identical. As a result, the inferred ZNF motifs from ChIP-seq data are significantly shorter than their intrinsic, full-length specificity profiles (11). With incomplete specificity information, it becomes impossible to accurately pinpoint the exact locations of their specific binding sites and distinguish relatively high-affinity binding sites from low-affinity ones, let alone understand their arms race with transposons.

Second, the ZNF ChIP-seq signals within repeat regions are often not quantitative enough for comparison between different repeat families. For instance, the vast majority of sequencing reads mapped onto the youngest LINE-1 member, L1HS, are multi-mapping reads (mapped to more than one genomic locus). Standard analysis pipelines, such as ENCODE (12), produce a ‘black hole’ within L1HS (Supplementary Figure S1), which happens to be the region of our interest. Generally, there are two ways to process multi-mapping reads: either remove them from downstream analysis or assign them to only one mapped locus randomly. Unfortunately, neither method is satisfactory for quantification purposes (13). Improved experimental and analytical strategies are necessary to address these two issues.

To tackle the first issue, Spec-seq (11,14) was developed to biophysically characterize the full-length specificity profiles of long ZNFs by examining a set of degenerate binding sites that cover all possible binding positions. The position energy matrix (PEM) derived from Spec-seq data provides quantitative, parametric information for predicting the binding energy of any full-length binding site with reasonably good accuracy (15), as long as the additivity assumption holds true for the studied TFs. Several human ZNFs, such as ZNF140, ZNF10, ZNF675, ZFP3 and ZNF382, have been successfully tested using Spec-seq, and their motifs are utilized in a separate ENCODE consortium project to validate the predictions of a machine learning algorithm that predicts genome-wide nucleotide importance (refer to Data Availability).

**Figure 1.**
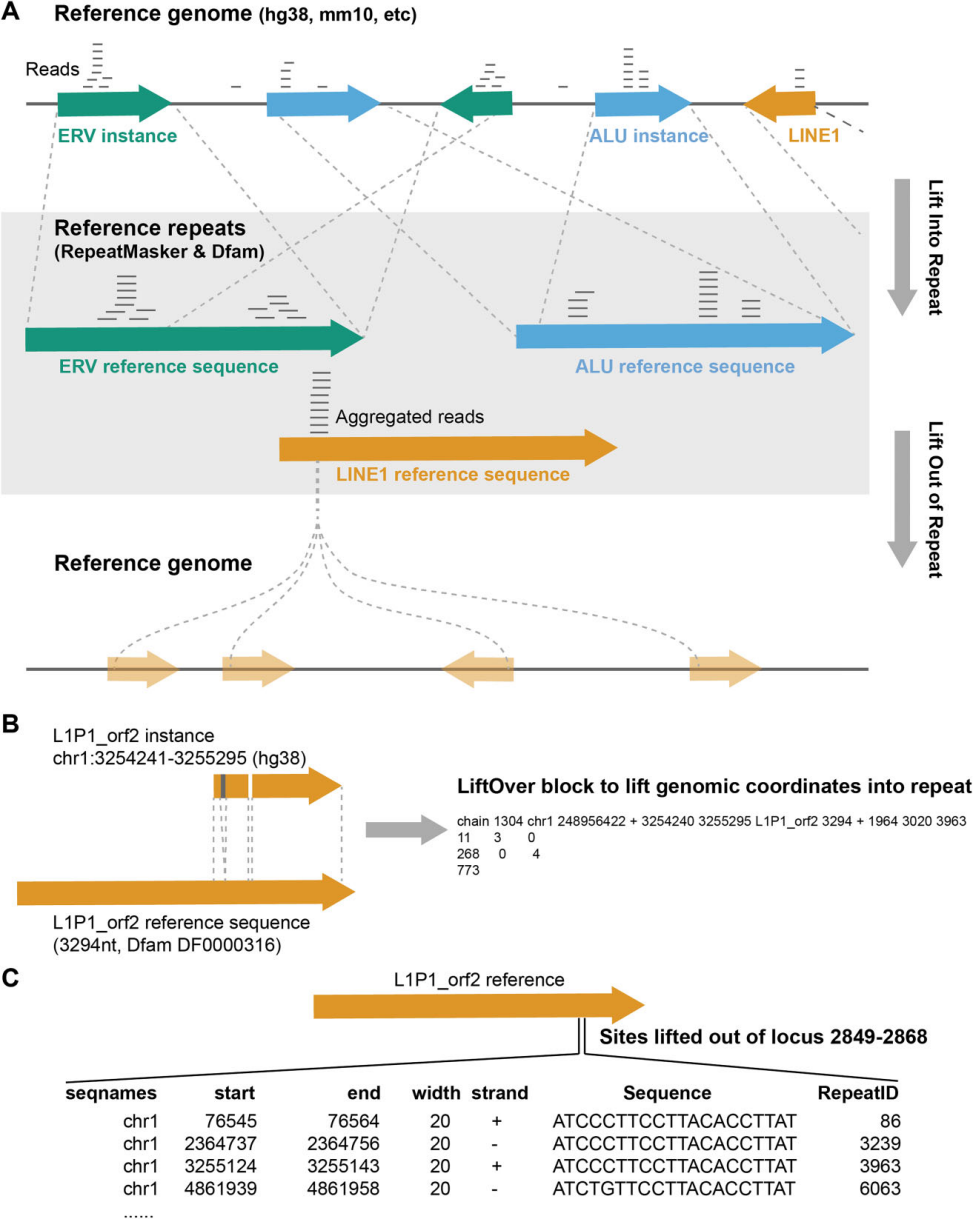
(**A**) Overall schematics for liftIn and liftOut operations of TECookbook; (**B**) Conversion of alignment file from RepeatMasker output to liftOver chain block for liftIn operation; (**C**) Example of liftOut operation to extract all genomic sites at defined repeat locus;

To address the second issue, Fernandes *et al.* (16) recently developed the UCSC Repeat Browser, which ingeniously maps ChIP-seq reads to reference repeat elements based on pairwise alignment between genomic sequences and reference repeat sequences through liftOver operation. This approach of lifting reads from genomic coordinates to corresponding reference repeat coordinates can be called ‘liftIn’ operation (Figure 1B). It is advantageous because each multi-mapping read is ultimately mapped to a unique repeat locus once, regardless of its initial genomic mapping, resulting in significantly improved signal-to-noise ratios. It should be pointed out that in their initial work, the ChIP-seq signals were displayed in as coverage profiles for ChIP-seq summits without any explicit normalization (Supplementary Figure S2), so it is unclear whether or not the signals shown on different reference repeats are comparable to each other, which could compromise our study of ZNFs’ silencing preference towards different repeat families. Appropriate signals normalization methods need to be tested and validated.

In addition to this liftIn operation and the intuitive GUI-based environment, a programming interface is needed to interact with other analysis protocols, as well as additional functions to quantify the degree of transposon evasion from ZNF across multiple repeat families. For example, the ability to extract all putative ZNF binding sites from any defined repeat locus and systematically evaluate/predict their binding affinity is essential (Figure 1C), which can be called ‘liftOut’ operation. Therefore, it is worthwhile to develop the R package TECookbook for these purposes. ZNF10 and ZNF382 are two known LINE-1 binders and their full-length specificity profiles have been biophysically characterized by Spec-seq, thus they serve as good examples here, not only to study the co-evolution between ZNFs and LINE-1 but also to justify the validity of the combined use of *in vivo* ChIP-seq data analysis through TECookbook and in vitro biophysical data for elucidating the functions of other long ZNFs in general.

## MATERIALS AND METHODS

TECookbook implements a method for mapping ChIP-seq reads, similar to the UCSC repeat browser approach. It first builds a liftOver chain file from RepeatMasker alignment data (.align file) and then lifts sequencing reads from genomic coordinates to reference repeat coordinates (Figure 1). However, there are two key differences.

First, to enable quantitative comparison of ChIP-seq signals across different reference repeat coordinates (e.g. L1P1 and L1P3), TECookbook also maps input control reads into reference repeat coordinates. This allows for the calculation of Fold Change over Control (F.C.C.) ratios at all positions with a prefixed window size (e.g. 10nt). In theory, the observed F.C.C. signal should indicate the average binding strength of the ZNF towards all sites within any defined repeat locus, regardless of repeat copy number. This makes it suitable for comparison purposes across different repeat families.

Second, TECookbook constructs the liftOver chain file strictly based on the alignment file (.align) provided by RepeatMasker, rather than using alternative alignment algorithms afterward. This ensures that the coordinates defined in reference repeats are fully consistent with the consensus sequences provided by the Dfam database (10). In addition to the liftIn operation, the liftOut operation facilitates the extraction of all sequences of any defined repeat locus when a RepeatMasker alignment file (.align) is provided, and three parameters (Repeat, Start, End) are specified.

For ChIP-seq analysis associated with human repeats, prebuilt annotation datasets can be directly downloaded from GitHub repository and Makefile for ZNF10 can serve as template to process ChIP-seq sequencing reads in general. For non-human data analysis, the buildChain and buildAnnotation functions are needed to construct the liftOver chain file and repeat annotation data first. UCSC liftOver tool is used to convert sequences data defined in genomic coordinates to data defined in reference repeat coordinates through the provided chain file.

Besides liftIn and liftOut operations, TECookbook package implements functions to annotate the ChIP-seq peaks or sets of binding sites based on their locations within or near repeats. The R package, code examples and analysis workflow including figures plotting can be accessed and reused through the GitHub repositories TECookbook and ZFPCookbook, as listed in the Supplementary Table S1 in the Supplementary Information.

## RESULTS

### TECookbook facilitates visualization of ZNF10 ChIP-exo signals across different LINE-1 sub-groups

**Figure 2.**
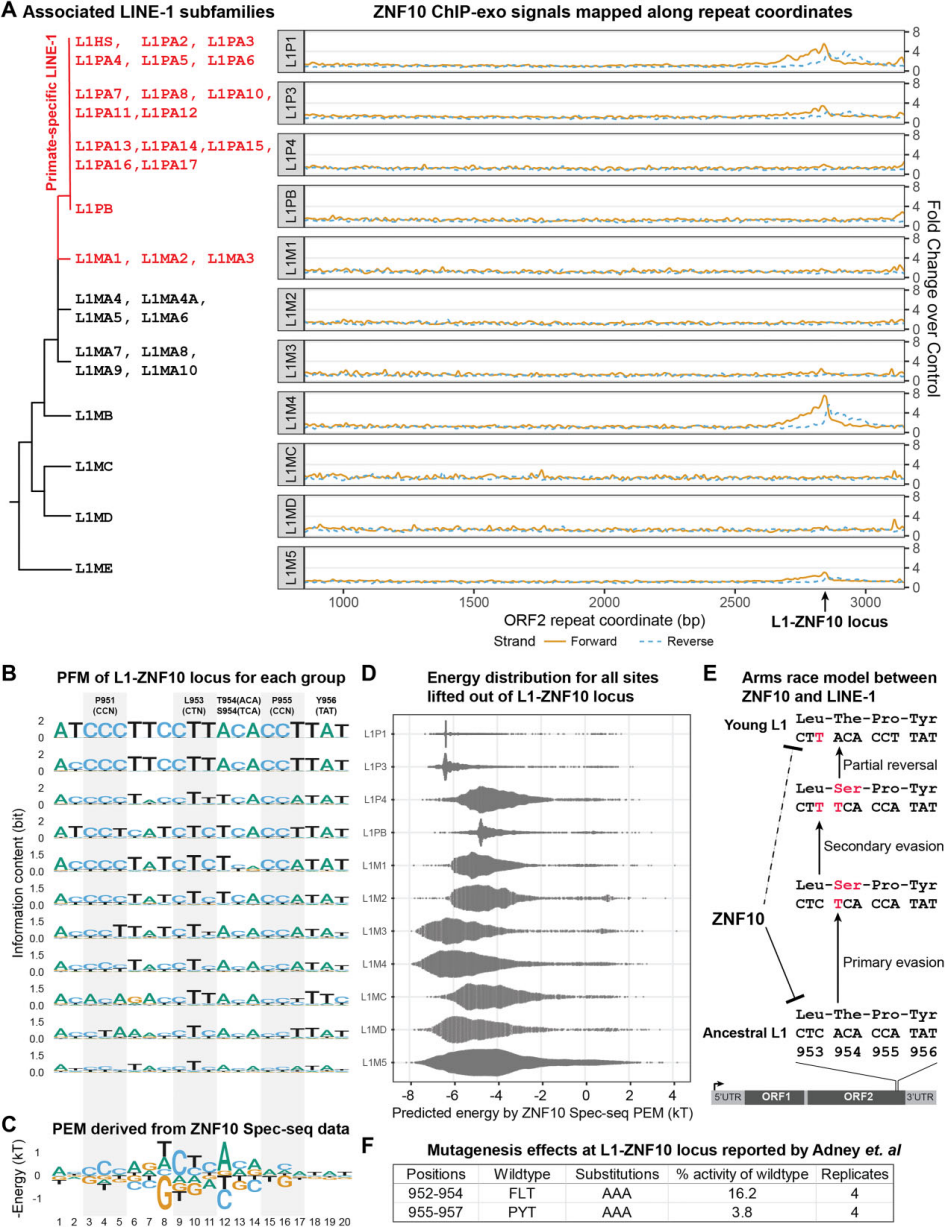
(**A**) ZNF10 ChIP-exo signals mapped onto LINE-1 ORF2 repeat coordinates; (**B**) PFM of L1-ZNF10 locus for each sub-group; (**C**) PEM derived from ZNF10 Spec-seq data; (**D**) Predicted energy distribution for all sites lifted out of each sub-group; (**E**) Arms race model between LINE-1 and ZNF10; (**F**) Mutagenesis effects at L1-ZNF10 locus (19).

LINE-1, the largest family of repeats in the human genome, has undergone multiple rounds of expansions, substitutions, and structural variations (17,18). Notably, its internal protein coding regions (ORF1 and ORF2) are more conserved than its 5′ and 3′ ends. In the standard RepeatMasker alignment (.align) file, the coding regions of closely related subfamilies are grouped together, such as L1P1_orf2, L1P3_orf2, L1PB_orf2, etc. Under this naming scheme, L1P1_orf2 includes the ORF2 coding regions of L1HS, L1PA2, L1PA3, L1PA4, L1PA5, L1PA6 together (Figure 2A’s left and right panels match horizontally). However, in the overall output (.out) file, each repeat element is classified as an individual LINE-1 subfamily based on its 5′ and 3′ sequence content (Figure 2A), despite some ambiguities. This discrepancy is well documented in the RepeatMasker database.

Among currently identified LINE-1 binders (2,16), ZNF10 is a conserved zinc finger protein (ZNF) found in at least 151 mammals (Source: NCBI gene database, not found in mouse). Comparison of human ZNF10 with putative orthologs from some representative species shows that its contact residues responsible for sequence recognition, remain highly conserved (Supplementary Figure S3), so we expect its binding specificity stayed the same along the whole mammalian lineage. To quantify its degree of silencing for different LINE-1 subfamilies, we can map all ChIP-exo reads to corresponding L1 orf2 coordinates and normalize them using control input reads, in either forward or reverse direction. Strong ChIP-exo signals are observed only in the L1-ZNF10 locus of L1P1, L1P3, L1M4 and L1M5 coordinates, with up to 8-fold enrichment over control (Figure 2A). This indicates that ZNF10 selectively targets certain LINE-1 subfamilies, including both old and young L1s. Strongest F.C.C. signals are observed in the L1M4 group, which is consistent with the fact that highest percentage of sites in L1M4 group (∼23%) contain intact CTCACA sequences shown by Spec-seq result at the core positions 9–14. It is important to note that the underlying repeat instances within each subgroup are distributed across the entire genome, so the observed ChIP-exo signals should be interpreted as the mean ChIP-exo signals of all included repeat instances.

### LINE-1 experienced at least two steps of evasion from ZNF10 and kept its retrotransposition activity

It was reported that LINE-1 escaped silencing by ZNF93 and ZNF765 through segmental deletion in its 5′ UTR region (4,16). But for ZNF10, the L1-ZNF10 locus is located within the ORF2 coding region (codon positions 950–956), meaning that any insertion or deletion would result in early termination and loss-of-function of the essential LINE-1 retrotransposase. Hypothetically, substitutions in certain key positions could be enough to enable L1-subfamilies to evade ZNF10.

To better understand ZNF10’s selective targeting, we can extract all potential ZNF10 binding sites from the L1-ZNF10 locus of each sub-group using the liftOut operation in TECookbook. We can then create a position frequency matrix (PFM) or sequence logo for each L1 sub-group (Figure 2B). For younger L1s, such as L1HS and L1PAs, the underlying sequences are quite similar, resulting in higher information content compared to older L1s.

Additionally, the position energy matrix (PEM) of ZNF10 can be reliably derived from Spec-seq data covering all possible positions in its 20-mer binding sites (Figure 2C). A visual comparison between the PFM of each L1 sub-group and ZNF10 Spec-seq PEM reveals that ORF2 codon positions 953-954, which match Spec-seq PEM positions 8-14 (TCTCACA), contribute the most to ZNF10’s preferential recognition of its underlying L1 response elements. Moreover, we can use Spec-seq PEM to quantitatively predict the binding energy of all sequence variants for each L1 sub-group, assuming an additive model is accurate for ZNF10 recognition. As expected, for ancestral L1s, most L1-ZNF10 loci contain significant substitutions or mismatches to the consensus, resulting in only a small fraction of L1-ZNF10 loci being potential high-affinity (low-energy) sites bound by ZNF10 (Figure 2D).

Two important observations are consistent with the ChIP-exo signals on repeat coordinates. First, the consensus sequences of the oldest L1 families, such as L1MB, L1MD and L1ME, align well with the core region of ZNF10 PEM. This strongly suggests that ZNF10 evolved in the mammal lineage to specifically silence ancestral L1 repeats. Second, L1 underwent at least two steps of substitutions in history to evade ZNF10 silencing, with only the primary evasion resulting in the codon change (T954S). Interestingly, the partial reversal (S954T) in recent primate-specific L1s has led to young L1s being recaptured by ZNF10 (Figure 2E). Adney et al. conducted systematic mutagenesis on L1 ORF2 and demonstrated that codons 952-957 are crucial for retrotransposase activity (19) (Figure 2F). It remains unclear whether the S954T restoration provides higher retrotransposition activity for recent primate L1s, despite the risk of being recaptured by ZNF10.

### ZNF382 is a broad-spectrum LINE-1 binder targeting a conserved region of ORF2

**Figure 3.**
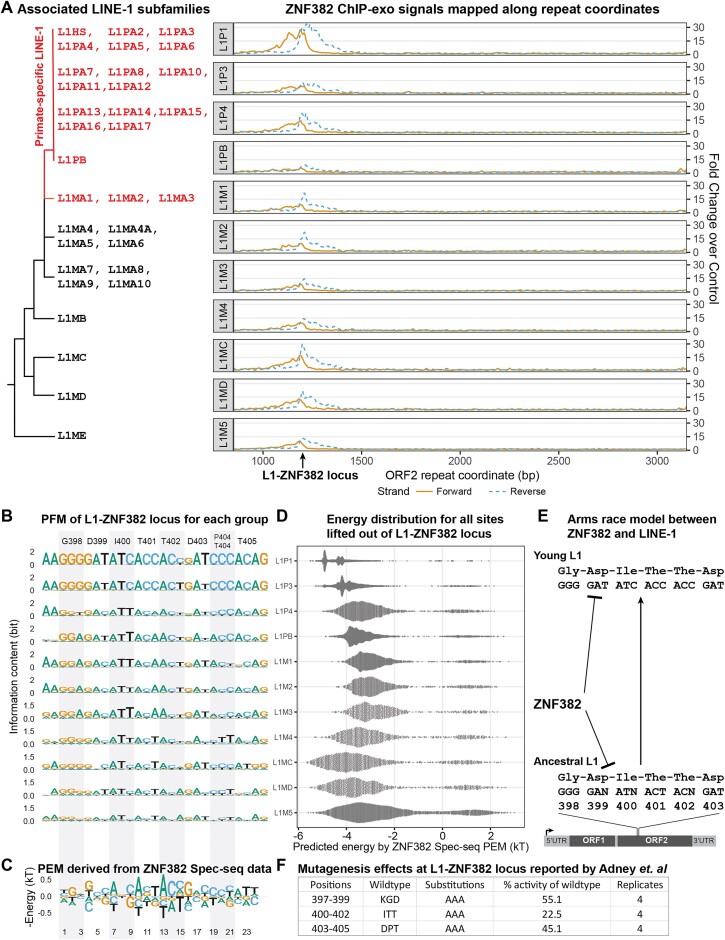
(**A**) ZNF382 ChIP-exo signals mapped onto LINE-1 ORF2 repeat coordinates; (**B**) PFM of L1-ZNF382 locus for each sub-group; (**C**) PEM derived from ZNF382 Spec-seq data; (**D**) Predicted energy distribution for all sites lifted out of each sub-group; (**E**) Arms race model between LINE-1 and ZNF382; (**F**) Mutagenesis effects at L1-ZNF382 locus (19).

**Figure 4.**
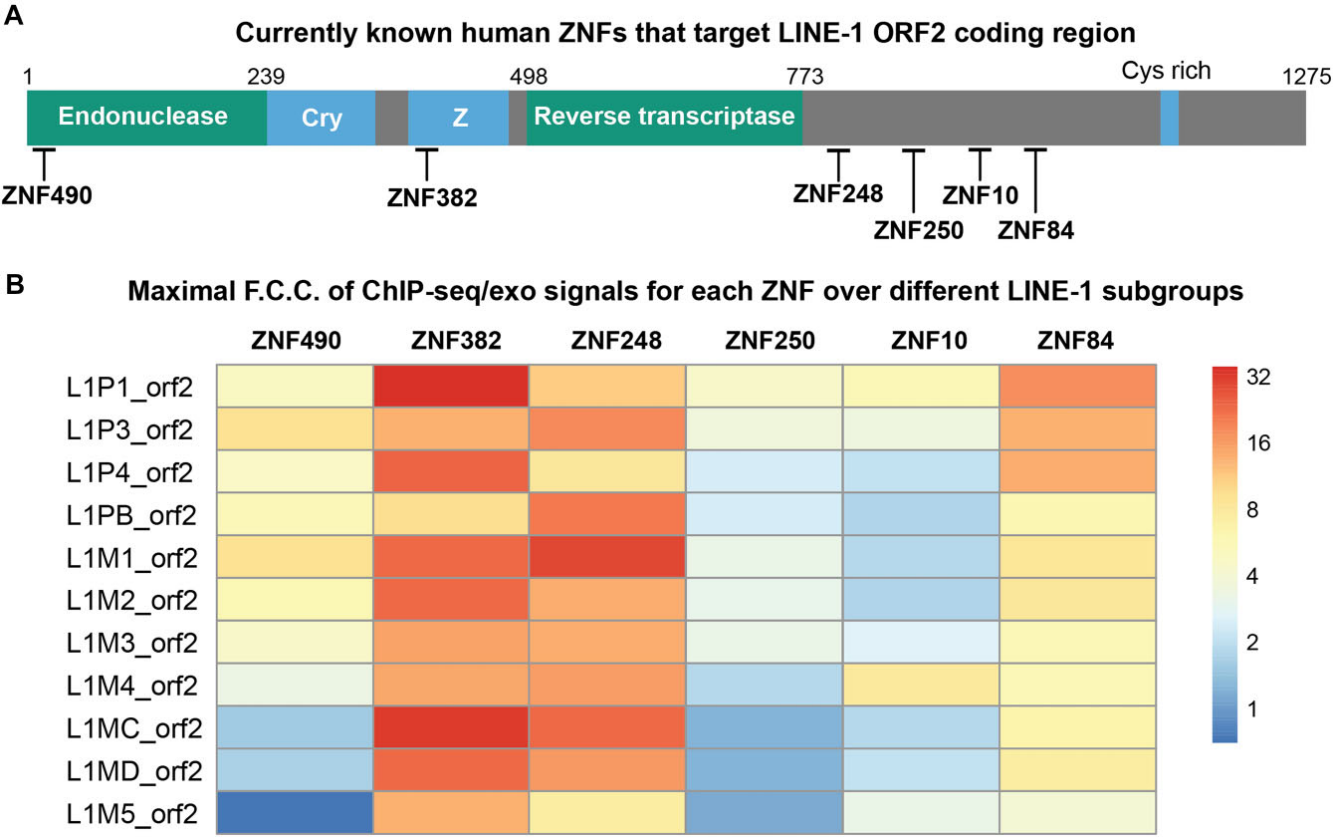
(**A**) The L1 ORF2 domain organization (adapted from Adney *et al.*) and some known ZNFs that target LINE-1; (**B**) Sub-group specificity of each ZNF based on maximal Fold Change over Control of ChIP-seq/exo data mapped on repeat coordinates.

ZNF382 is another KRAB-ZNF that targets the LINE-1 ORF2 region and has been characterized by Spec-seq. Because of the conserved contact residues compositions for its orthologs identified by NCBI in mammals (Supplementary Figure S3), we expect its intrinsic specificity also stayed the same along mammalian lineage. Unlike other LINE-1 repressors such as ZNF93, ZNF10 and ZNF765, ZNF382 is unique in that it targets all subfamilies of LINE-1 (Figure 2A). Its binding sites, the L1-ZNF382 locus, encode the L1 ORF2 protein positions 398-405, which are situated between the N-terminal endonuclease and C-terminal reverse transcriptase domain (Figure 4A) and have been shown to be important for overall retrotransposition activity (Figure 3F). Comparing the PEM derived from Spec-seq data (Figure 3C), it is evident that both old and young L1s match the intrinsic specificity of ZNF382 quite well. Strongest ZNF382 F.C.C. signals are observed in the L1P1 and L1MC groups, which is consistent with the fact that highest percentage of sites in these two groups (48.7% and 14.2% respectively) have binding energy below –4.5kT predicted by the Spec-seq PEM (Figure3C, D). However, intermediate-age groups from L1PB to L1M4 contain mismatches at positions 9 and 15 without codon changes, which is consistent with their relatively high energy distribution (Figure 3D) and low ChIP-exo signals (Figure 3A). Unless there is some other fitness benefit for LINE-1 to be persistently targeted by ZNF382, current data suggests that synonymous substitutions in the L1-ZNF382 locus are not enough for LINE-1 to completely evade ZNF382 repression.

In addition to ZNF10 and ZNF382, at least four other ZNFs have been identified as LINE-1 binders targeting the ORF2 coding region (16) (Figure 4A). Therefore, it is feasible to map their ChIP-seq/exo signals onto reference repeat coordinates and compare their sub-group preference (Supplementary Figures S4–S6). ZNF248 has been found to be another broad-spectrum LINE-1 binder (Figure 4B). Both ZNF382 and ZNF248 are specifically upregulated in neuronal and glial cells (Supplementary Figure S7) (20), suggesting that effective LINE-1 repression in the nervous system may require the engagement of multiple human ZNFs.

## DISCUSSION

Compared to the UCSC Repeat Browser, TECookbook is designed to quantify and compare ZNF binding profiles across different repeat families by normalizing against input controls, rather than only displaying coverage profiles for ChIP-seq summits without proper normalization (Supplementary Figure S2). The resulting F.C.C. signals can be well explained by the underlying binding site strength predicted by ZNF382 Spec-seq data, which supports its general use to understand the binding preference of other transposon repressors. Sun *et al.* (21) implemented alternative approach to map the ChIP-seq signals onto L1 repeats, but in their initial paper only L1HS repeats were analyzed in detail and the role of KRAB-ZNF wasn’t discussed. Besides mapping ChIP-seq/exo signals through liftIn operation, TECookbook implements the liftOut function to extract all putative binding sites in a given repeat locus for estimating binding energy using a PEM derived from in vitro experiments like Spec-seq or HT-SELEX. It can also be used to annotate the repeat associations of ChIP-seq peaks or binding sites. In the long run, more functions will be added for parsing and annotating repeat-associated data in collaboration with the broader community.

The arms race models of ZNF10 and ZNF382, based on the current analysis, suggest that besides structural variation, nucleotide substitution is a common mechanism of transposon evasion from ZNFs (6). To date, no broad-spectrum LINE-1 repressor has been found to target the 5'UTR or promoter regions of LINE-1. The necessity of maintaining retrotransposase activity imposes strong constraints on the possible type (substitution or indel) and form (synonymous or non-synonymous) of repeat evasions in the protein coding regions. As a result, it could be easier for ZNFs to achieve broad-spectrum, persistent silencing by targeting protein-coding regions rather than non-coding regions. The almost identical contact residues composition of ZNF10 and ZNF382 among mammals suggests that there exists some selective pressure to maintain their intrinsic specificity profiles and biological functions (Supplementary Figure S3). Recent GWAS analysis from the UK Biobank (22) showed that the loss-of-function of ZNF10 and ZNF382 is associated with a high risk of headache and corneal hysteresis, respectively, although it is unclear whether these are direct consequences of derepressed expression of LINE-1 transcripts.

It is very likely that many ZNFs have targeted transposons throughout evolutionary history, but as the targeted transposons are no longer active, those ZNFs have been co-opted for alternative biological functions (23,24). Understanding the specificity profiles of these long ZNFs through *in vitro* biophysical experiments, such as Spec-seq, remains helpful for deciphering their functions today.

## Supplementary Material

lqad078_Supplemental_File

## Data Availability

Data and analysis workflows are listed in Supplementary Table S1 in Supplemental Information. TECookbook package is freely available for re-use under the GPL license through GitHub repository TECookbook and DOI: 10.5281/zenodo.8016038. The Spec-seq data of ZNF10 and ZNF382 was generated while Z.Z. worked as postdoc in Fordyce lab at Stanford to validate the correctness of a machine learning algorithm developed by Kundaje lab as part of one ENCODE consortium project and has been deposited at NCBI GEO database (GSE189817) for public access. Personal communication with Prof. Ansul Kundaje is suggested if one has question about that separate project.
