# Supplementary material for: Quantifying the arms race between LINE-1 and KRAB-zinc finger genes through TECookbook
Source: NAR Genom Bioinform. 2023 Sep 6;5(3):lqad078. doi: 10.1093/nargab/lqad078 (PMC10480687; doi:10.1093/nargab/lqad078)
Supplement: lqad078_Supplemental_File [file lqad078_supplemental_file.pdf]

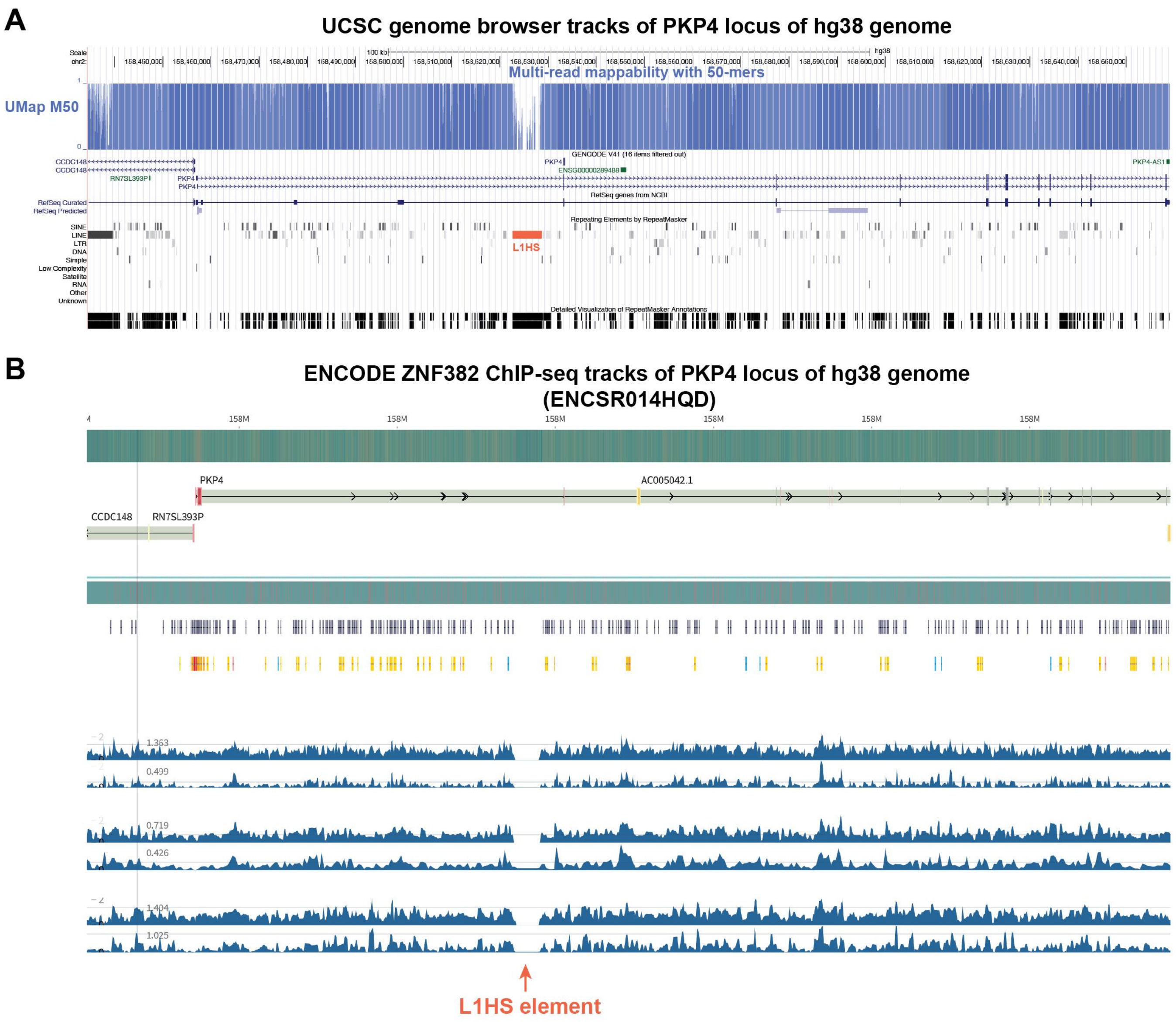

Figure S1

A) UCSC genome browser tracks of PKP4 locus of human genome;

B) ZNF382 ChIP-seq tracks of PKP4 locus produced and analyzed by ENCODE pipeline;

UCSC repeat browser tracks for ZNF10 and ZNF382 in L1HS region

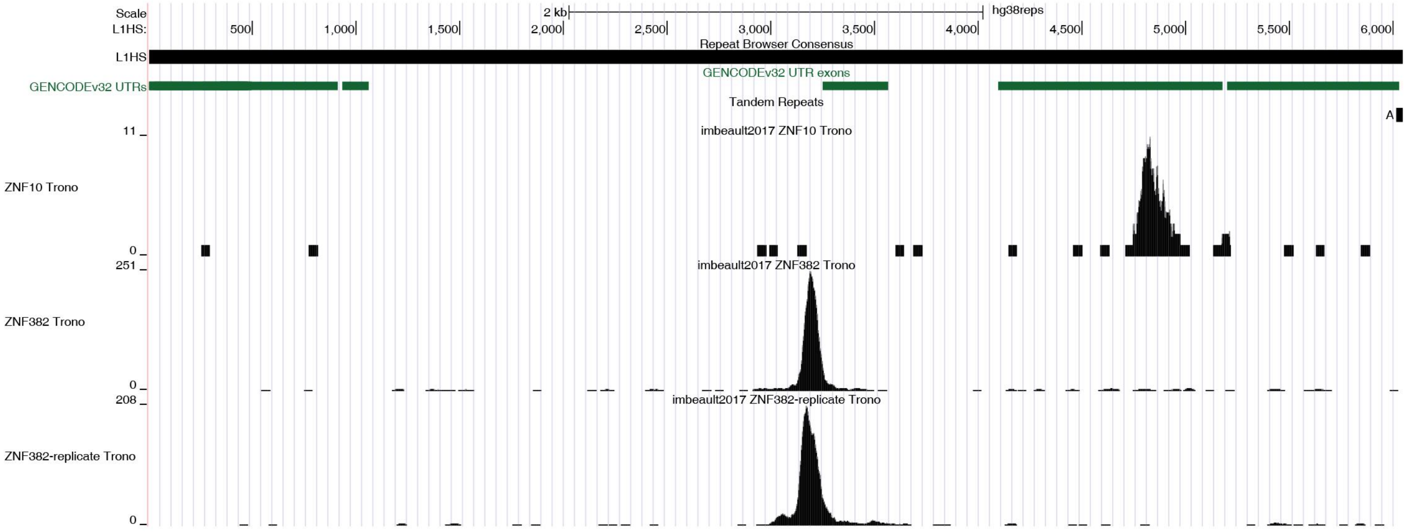

UCSC repeat browser tracks for ZNF10 and ZNF382 in L1P3 region

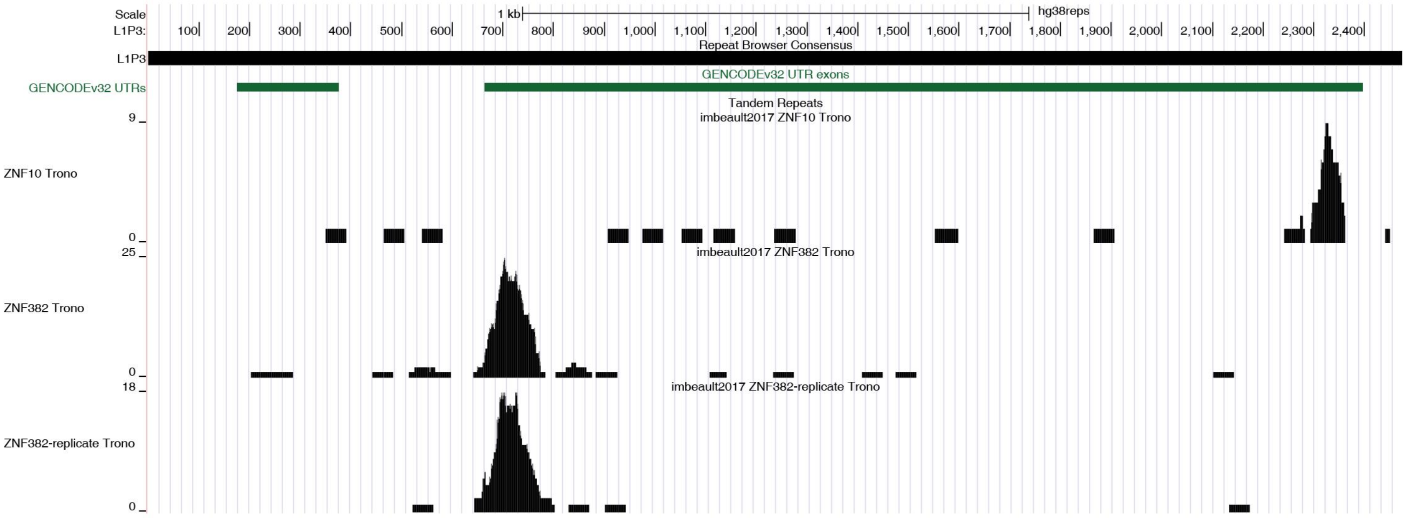

UCSC repeat browser tracks for ZNF10 and ZNF382 in L1M4 region

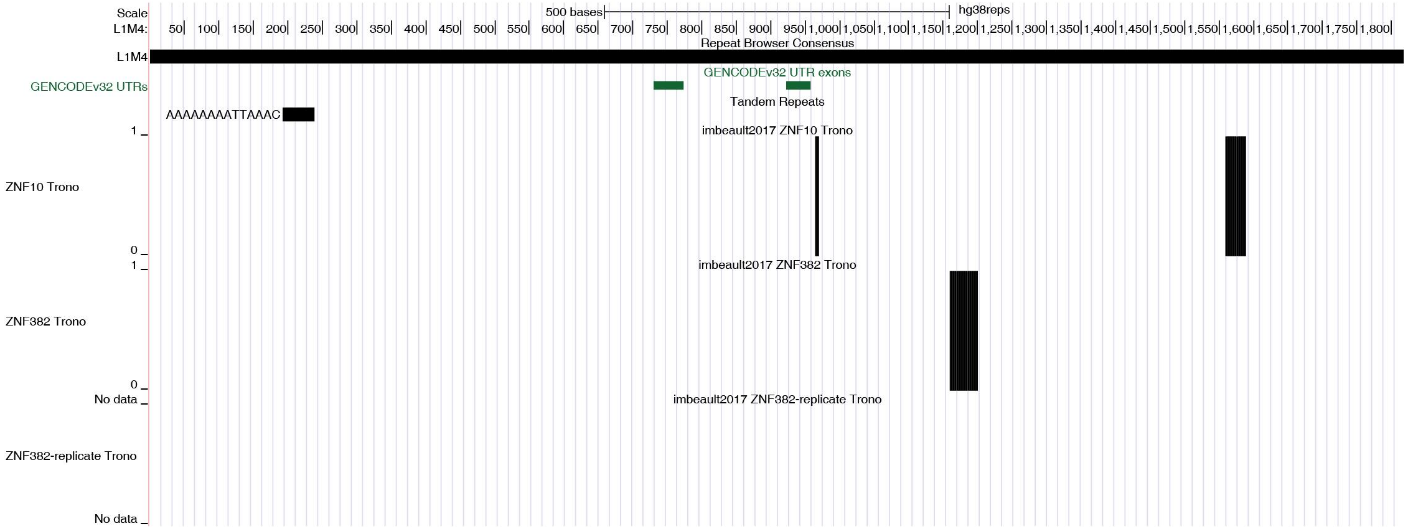

Fig. S2 UCSC repeat browser tracks for ZNF10 and ZNF382 in different LINE-1 repeats (L1HS, L1P3 and L1M4)

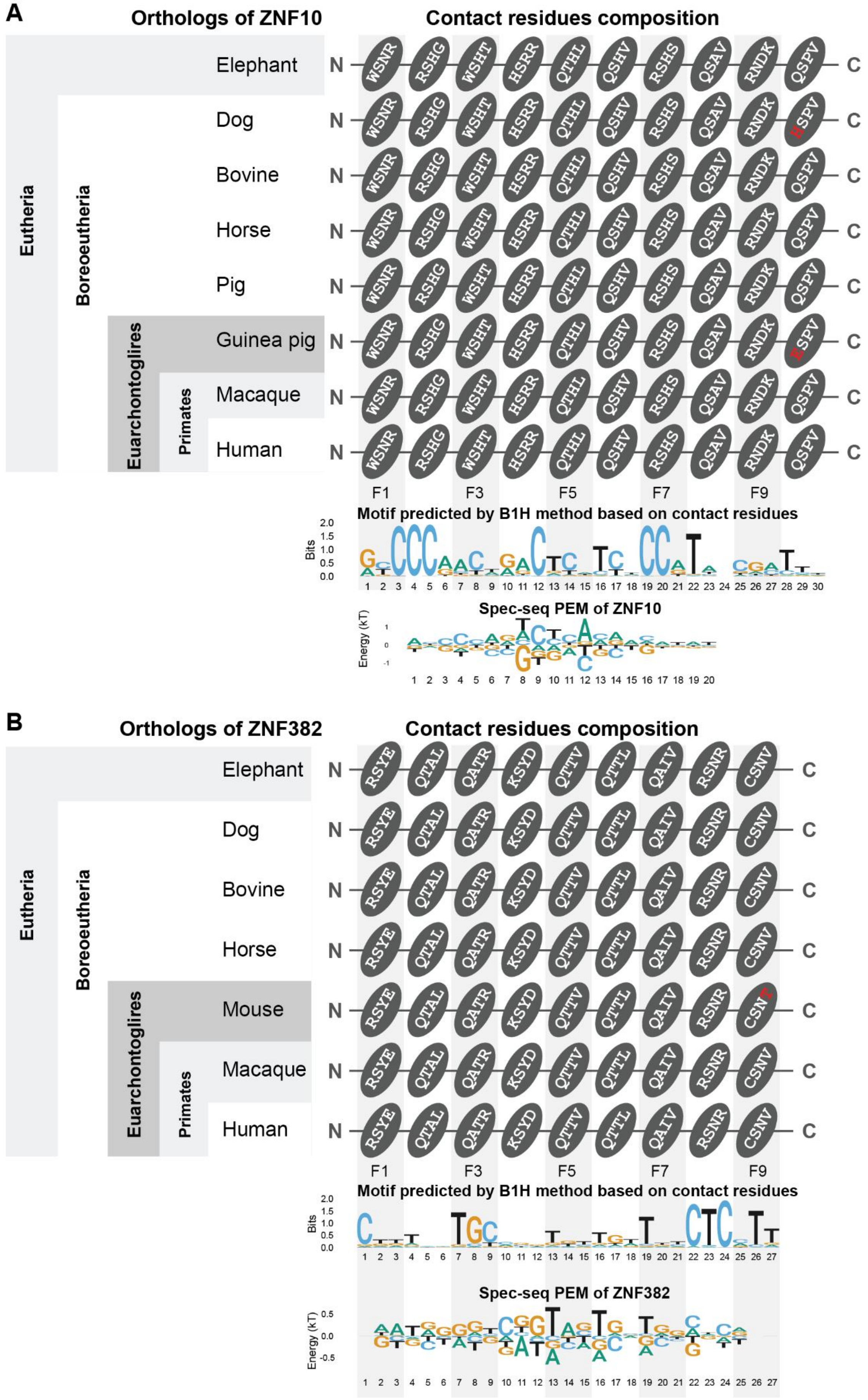

Figure S3 Contact residues composition and predicted motifs of ZNF10 and ZNF382 respectively. (Protein sequences source: NCBI gene database)

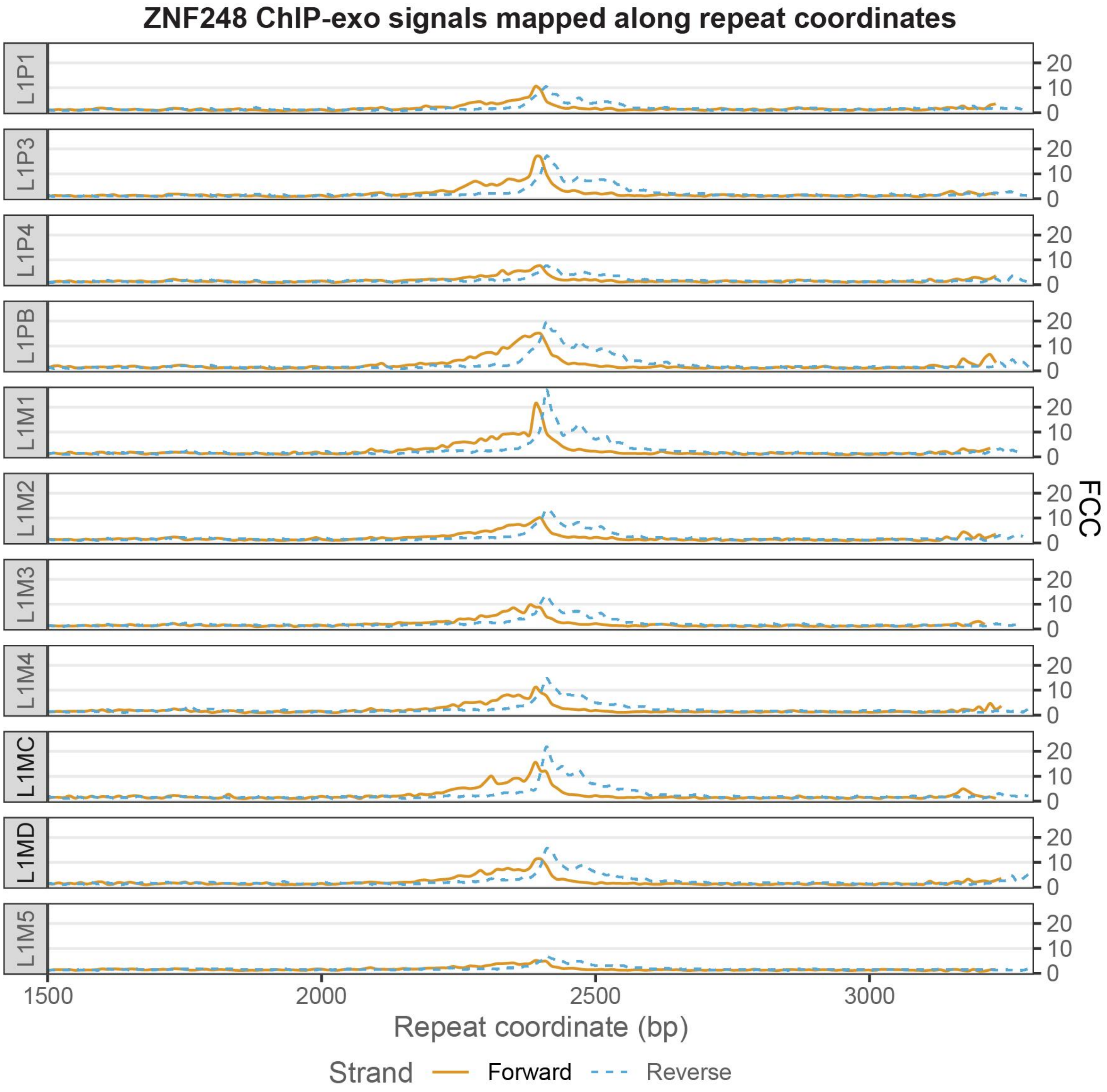

Figure S4 ZNF248 ChIP-exo signals mapped onto LINE1-ORF2 repeat tracks

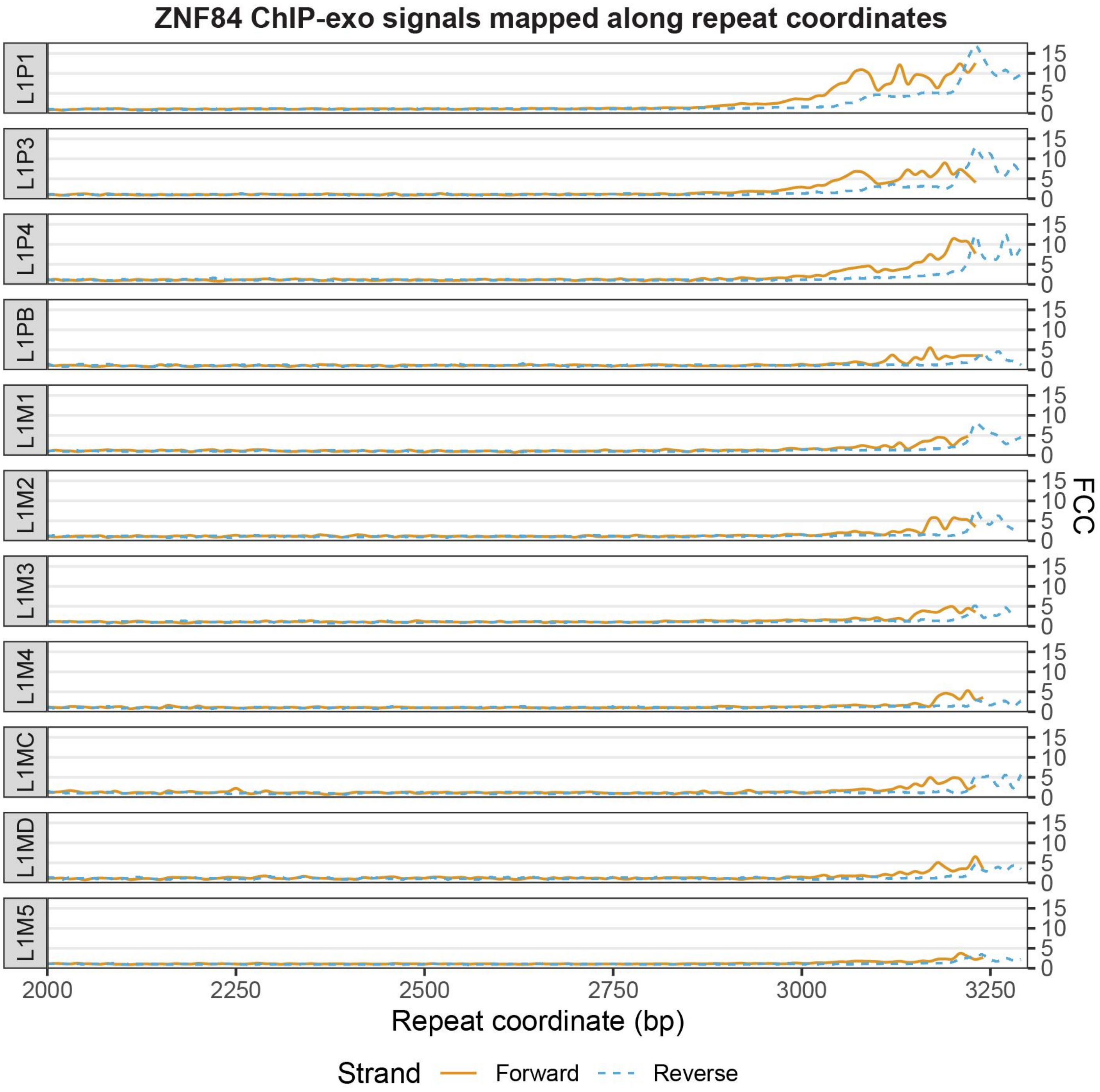

Figure S5 ZNF84 ChIP-exo signals mapped onto LINE1-ORF2 repeat tracks

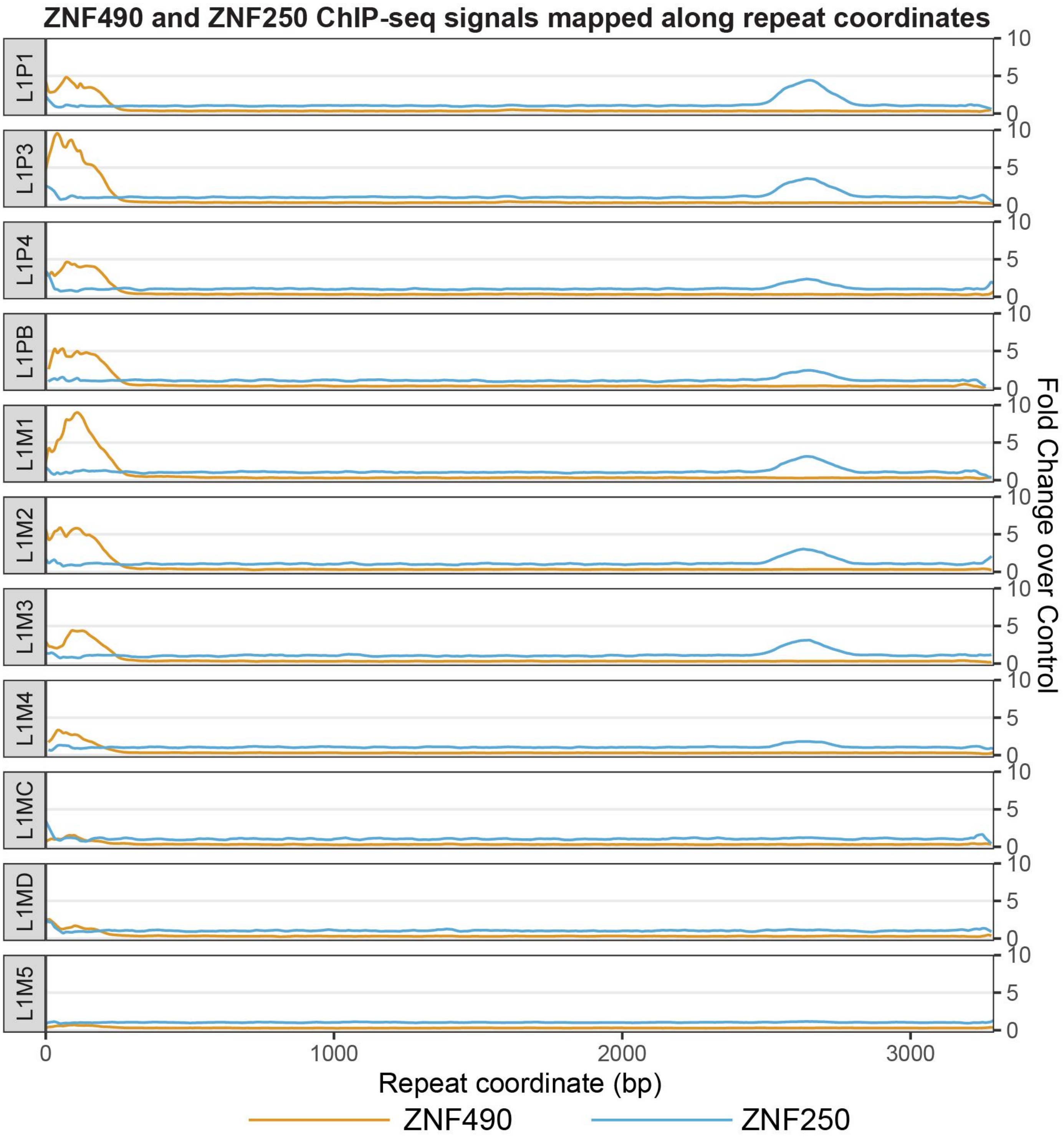

Figure S6 ZNF490 and ZNF250 ChIP-seq signals mapped onto LINE1-ORF2 repeat tracks

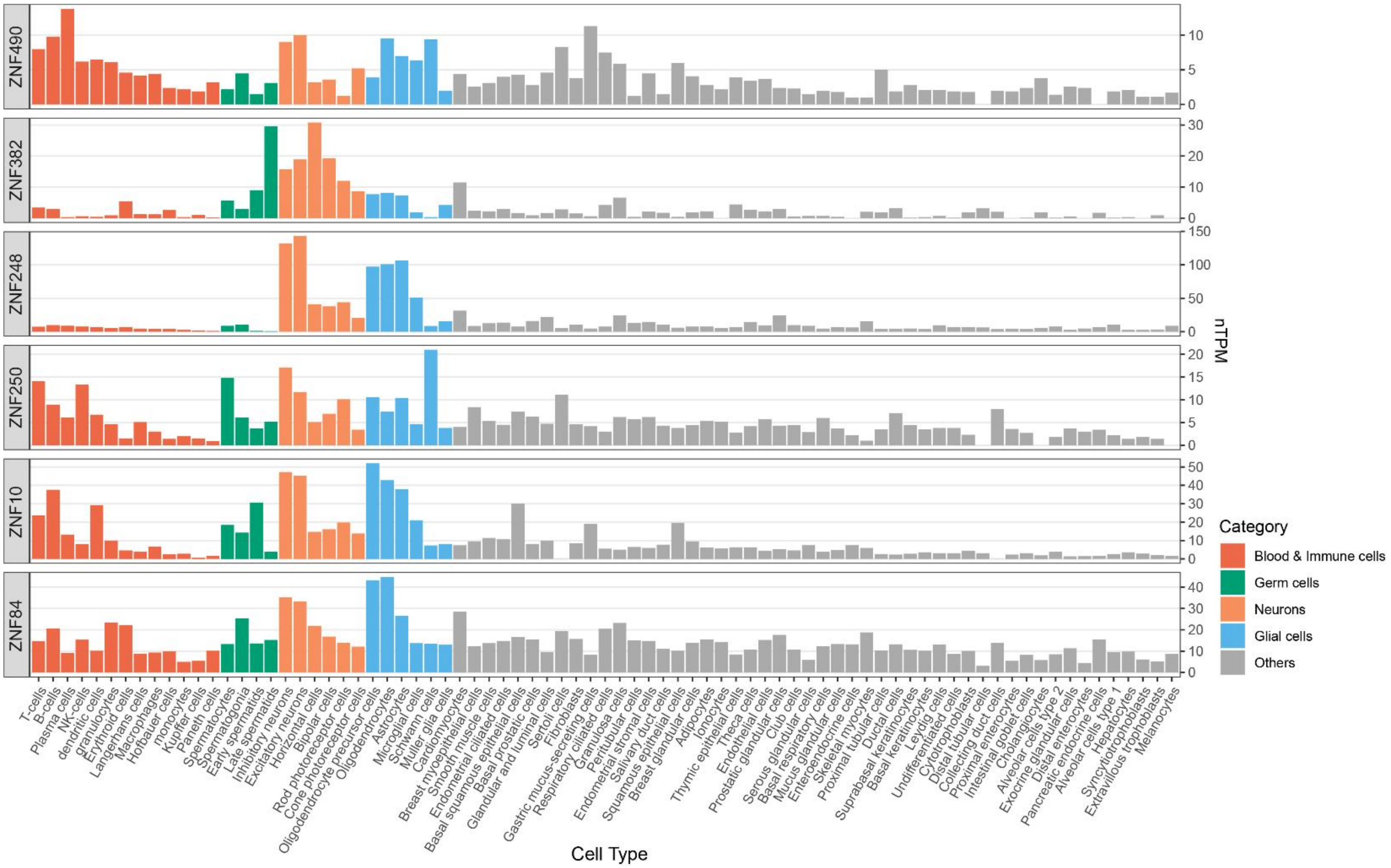

Figure S7 Expression levels of ZNFs over human cell types by Human Protein Atlas

REAGENT or RESOURCE	SOURCE	ACCESS IDENTIFIER
**Software, Algorithms, and Analysis workflow**		
MEME	MEME suite	http://meme-suite.org/
TECookbook	Zheng Zuo	https://github.com/zeropin/TECookbook
TFCookbook	Zheng Zuo	https://github.com/zeropin/TFCookbook
Analysis of ZNF10 and ZNF382	Zheng Zuo	https://github.com/zeropin/ZFPCookbook
**Data source**		
ZNF10 and ZNF382 Spec-seq data	Zheng Zuo	NCBI GEO GSE189817
Zinc finger motif database	Hughes Lab	http://kznfmotifs.ccbr.utoronto.ca/
ChIP-exo data for ZNF10, 84, 248, and 382	Trono lab	NCBI GEO GSE78099
ChIP-seq data for ZNF250 and ZNF490	Hughes Lab	NCBI GEO GSE76496
Expression levels of ZNF382 and ZNF248	Human Protein Atlas	https://www.proteinatlas.org
ZNF genes association with phenotypes	GeneBass	Genebass.org
UCSC repeat browser	UCSC	https://repeatbrowser.ucsc.edu/

Table S1 Key resources table
